# The Repetitive Cytoskeletal Protein H49 of *Trypanosoma cruzi* Is a Calpain-Like Protein Located at the Flagellum Attachment Zone

**DOI:** 10.1371/journal.pone.0027634

**Published:** 2011-11-11

**Authors:** Alexandra Galetović, Renata T. Souza, Marcia R. M. Santos, Esteban M. Cordero, Izabela M. D. Bastos, Jaime M. Santana, Jeronimo C. Ruiz, Fabio M. Lima, Marjorie M. Marini, Renato A. Mortara, José Franco da Silveira

**Affiliations:** 1 Departamento de Microbiologia, Imunologia e Parasitologia, Escola Paulista de Medicina, Universidade Federal de São Paulo, São Paulo, São Paulo, Brasil; 2 Laboratorio de Bioquímica, Departamento Biomédico, Universidad de Antofagasta, Antofagasta, Chile; 3 Universidade Bandeirante, São Paulo, São Paulo, Brasil; 4 Departamento de Biologia Celular, Instituto de Biologia, Universidade de Brasilia, Brasilia, Brasil; 5 Centro de Pesquisa René Rachou (CPqRR), FIOCRUZ, Belo Horizonte, Minas Gerais, Brasil; University of Georgia, United States of America

## Abstract

**Background:**

*Trypanosoma cruzi* has a single flagellum attached to the cell body by a network of specialized cytoskeletal and membranous connections called the flagellum attachment zone. Previously, we isolated a DNA fragment (clone H49) which encodes tandemly arranged repeats of 68 amino acids associated with a high molecular weight cytoskeletal protein. In the current study, the genomic complexity of H49 and its relationships to the *T. cruzi* calpain-like cysteine peptidase family, comprising active calpains and calpain-like proteins, is addressed. Immunofluorescence analysis and biochemical fractionation were used to demonstrate the cellular location of H49 proteins.

**Methods and Findings:**

All of H49 repeats are associated with calpain-like sequences. Sequence analysis demonstrated that this protein, now termed H49/calpain, consists of an amino-terminal catalytic cysteine protease domain II, followed by a large region of 68-amino acid repeats tandemly arranged and a carboxy-terminal segment carrying the protease domains II and III. The H49/calpains can be classified as calpain-like proteins as the cysteine protease catalytic triad has been partially conserved in these proteins. The H49/calpains repeats share less than 60% identity with other calpain-like proteins in *Leishmania* and *T. brucei*, and there is no immunological cross reaction among them. It is suggested that the expansion of H49/calpain repeats only occurred in *T. cruzi* after separation of a *T. cruzi* ancestor from other trypanosomatid lineages. Immunofluorescence and immunoblotting experiments demonstrated that H49/calpain is located along the flagellum attachment zone adjacent to the cell body.

**Conclusions:**

H49/calpain contains large central region composed of 68-amino acid repeats tandemly arranged. They can be classified as calpain-like proteins as the cysteine protease catalytic triad is partially conserved in these proteins. H49/calpains could have a structural role, namely that of ensuring that the cell body remains attached to the flagellum by connecting the subpellicular microtubule array to it.

## Introduction

The flagellum of the parasitic protozoan *Trypanosoma cruzi*, the etiological agent of Chagas disease, is a complex and specialized structure with critical roles in motility, cellular division and morphogenesis. It differs from its counterparts in mammalian cells in several structural, biochemical and immunological respects, suggesting that its components may be potential targets for the development of new anti-parasitic drugs. Several *T. cruzi* flagellar and cytoskeletal proteins are potent immunogens in humans and have been used as specific diagnostic and prognostic antigens in the serodiagnosis of Chagas' disease [1], [2], [3]. Immunization of mice with purified or semi-purified fractions of *T. cruzi* cytoskeleton induced high levels of specific humoral and cellular immune responses that protected the mice against a fatal challenge [4], [5], [6].

Trypanosomes have a single flagellum that emerges from the flagellar pocket and remains attached along the cell body for most of its length, with the exception of the distal tip. This adhesion region, named the flagellar attachment zone (FAZ), is a complex system of membrane connections, filaments and specialized microtubules [7], [8], [9], [10], [11]. At the cytoplasmic side there is an electron-dense filament and a quartet of microtubules connected to the smooth endoplasmic reticulum; both lie below the plasma membrane and follow the flagellum length. Several studies have suggested that the FAZ region plays a role in cellular organization and cytokinesis [12], [13]. FAZ structures are replicated and associated with the new flagellum.

The FAZ region has been well characterized at the morphological level, but the majority of its components are as yet unknown. The molecular characterization of these components is not an easy task, as several are found at very low levels and are often insoluble. In *T. cruzi*, a few proteins have been identified in the FAZ region. The membrane glycoprotein of 72 kDa (GP72) is concentrated in the FAZ region, and distributed over the surface of the cell body and the flagellar pocket membrane [14], [15]. *T. cruzi* GP72 null mutants have an unexpected morphology characterized by the detachment of the flagellum from the cell body, leading to major alterations in the overall shape of the parasite [14], [15]. The FAZ1 protein was identified in *T. brucei* using the monoclonal antibody L3B2, and it is required for normal FAZ assembly and flagellum attachment [16]. In *T. cruzi* epimastigotes, L3B2 antibody reacted with the initial portion of the flagellar-cell body adhesion zone, suggesting the presence of a *T. cruzi* FAZ1 homologue [10]. Several *T. cruzi* antigens have been isolated by screening genomic and cDNA expression libraries with sera from human Chagasic patients or infected animals [1], [17], [18], [19], [20], [21], [22]. One such antigen, H49, encodes a high molecular mass repetitive protein, composed of 68-amino acid repeats tandemly arranged [17], [23]. Immunoelectron microscopy demonstrated that H49 is located along the attachment region between the flagellum and the cell body [17]. Another FAZ component was identified using the monoclonal antibody 4D9; it reacted with a high molecular weight protein located in the cell body of the FAZ region [10], [24], [25].

In this study, the structure of the H49 protein and its repeats is further characterized. Sequence analysis demonstrated that the 68-aa repeats are located in the central domain of calpain-like cysteine peptidases, suggesting that H49 proteins are members of a novel family of calpain-related genes in *T. cruzi*. According to domain structure and sequence composition, calpain-like proteins in trypanosomatids were classified into five groups (I to V) [26]. Our studies have focused on proteins belonging to group IV, which contain the second and third domains separated by varying numbers of tandem amino acid repeats. Critical alterations in the Cysteine-Histidine-Asparagine (CHN) catalytic motif suggest that H49 proteins lack calpain proteolytic activity and are non-enzyme homologues. The sub-cellular location of H49 proteins in the FAZ region were identified using biochemical and immunofluorescence analyses, and suggest a distinct role for these proteins in *T. cruzi*.

## Methods

### Ethics Statement

This study was carried out in strict accordance with the recommendations in the Guide for the Care and Use of Laboratory Animals of the National Institutes of Health. The protocol was approved by the Committee on the Ethics of Animal Experiments of the Federal University of Sao Paulo (Permit Number: CEP09555-07). All surgery was performed under sodium pentobarbital anesthesia, and all efforts were made to minimize suffering.

#### Parasites

*T. cruzi* clone CL Brener [27], [28] and strain G [29] were used in this study. Parasites were maintained by cyclic passage in mice and in axenic cultures in liver infusion tryptose medium containing 10% fetal calf serum at 28°C.

#### Southern blot analysis and pulsed-field gel electrophoresis

DNA samples isolated from epimastigotes as previously described [30] were digested with restriction enzymes, separated by electrophoresis on agarose gels (0.8%) and stained with ethidium bromide (0.5 µg/mL). They were incubated with 0.25 M HCl for 45 min, denatured with 0.5 M NaOH/1 M NaCl for 20 min, neutralized with 1 M Tris-base/0.5 M NaCl for 20 min and transferred to nylon membranes in 20X SSC (1X SSC =  0.15 M NaCl/0.015 M sodium citrate); DNA was fixed by exposure to 150 mJ of UV radiation in a GS Gene Linker UV chamber (Bio-Rad). The membranes were prehybridized in a solution containing 50% formamide/5X SSC/5X Denhardt's solution (Invitrogen)/0.1 mg/mL salmon sperm DNA/0.1 mg/mL tRNA at 42°C for 2 h and hybridized overnight at 42°C with α^32^P-labeled probes, consisting of DNA fragments corresponding to various regions of the H49/calpain genes. Following hybridization, the membranes were subjected to two washes (30 min each at 42°C) with 2X SSC containing 0.1% SDS, one wash (30 min at 42°C) with 1X SSC containing 0.1% sodium pyrophosphate and one additional wash at 56°C with 0.1X SSC containing 0.1% SDS/0.1% sodium pyrophosphate. They were exposed to X-ray film thereafter.

*T. cruzi* chromosomal DNA was separated by pulsed-field gel electrophoresis in a Gene Navigator System (Amersham Pharmacia Biotech, NJ, USA), using a hexagonal electrode array. PFGE was carried out in 1.2% agarose gels in 0.5X TBE (45 mM Tris/45 mM boric acid/1 mM EDTA, pH 8.3) at 13°C for 132 h as previously described [31]. Gels were stained with ethidium bromide, photographed, transferred to nylon filters, and hybridized as described above.

#### Expression and purification of GST-H49, GST-CysPc domain and GST-H49 degenerated/calpain recombinant proteins

The 204-bp repeat of the H49 gene was cloned in a pGEX vector (GE Healthcare) as described previously [32]. The catalytic domain (CysPc) and the H49 degenerated repeats (H49 deg) were amplified by PCR with specific primers based on the H49/calpains XM_799896 and XM_804900, respectively (Table S2). All nucleotide sequences were cloned in frame with glutathione S-transferase (GST) in a pGEX-3X vector (GE Healthcare). GST-CysPc and GST-H49 recombinant plasmids were transformed into *Escherichia coli* DH-5α, and GST-H49 deg into *E. coli* BL21 (DE3). After growing in LB medium and being induced with 1 mM isopropyl-β-D-thiogalactopyranoside for 4 h, the transformed bacteria were washed with PBS, resuspended in 20 mL PBS containing 1% Triton X-100/4 mg/mL lysozyme/1 mM PMSF, incubated for 10 min at 4°C, sonicated and centrifuged at 17,210 x *g* for 15 min at 4°C.

The GST-H49 recombinant protein was affinity purified from bacterial lysates using a prepacked glutathione Sepharose 4B column (Amersham Pharmacia Biotech). The yield and purity were checked by protein concentration measurement and SDS-PAGE, respectively.

The recombinant proteins GST-CysPc and GST-H49 deg were purified from polyacrylamide gels. Briefly, the gels were treated with ice-cold 250 mM KCl in order to precipitate the SDS and visualize the proteins bands. The expected size bands were excised, frozen and shattered. The mixture was eluted for 16 h under constant agitation at room temperature in five volumes of solution containing 50 mM Tris-HCl (pH 8.0)/5 mM EDTA/1 mM PMSF/50 mM NaCl. Two separate approaches were adopted to remove the SDS, which remains attached to the proteins, from elutes. The GST-CysPc elute was dialyzed against two exchanges of 10 mM ammonium bicarbonate for 16 h at 4°C followed by two exchanges of bidistilled water for 16 h at 4°C and two exchanges of PBS buffer for 16 h at 4°C. The dialyzed volume was concentrated against sucrose. GST-H49 deg elutes (100 µL) were combined with 400 µL methanol, 100 µL chloroform and 300 µL of bidistilled water, and homogenized for 15 s. The mixture was incubated at −70°C for two min and centrifuged at 10,300 x *g* for 10 min. The protein interface was recovered, washed with 300 µL methanol and centrifuged at 10,300 x *g* for 5 min. The pellet was washed with 500 µL methanol: acetone (1∶1) and centrifuged at 10,300 x *g* for 5 min. The recombinant protein was resuspended in 50 mM Tris-HCl (pH 8.0) and stored at −20°C [33].

#### Production of anti-H49, anti-CysPc domain and anti-H49 deg antibodies

Anti-H49, anti-CysPc domain and anti-H49 deg polyclonal antibodies were generated by intraperitoneal immunization of BALB/c mice with four doses of recombinant protein (30 µg/mouse) and Al(OH)_3_ as an adjuvant (3 mg/mouse) at 7-day intervals. Seven days after the last immunizing dose, animals were bled and the sera were stored at −20°C. In addition, anti-H49, anti-CysPc domain and anti-H49 deg polyclonal antibodies were generated by immunizing New Zealand rabbits with four doses of recombinant protein (100 µg/rabbit). The first inoculation was with complete Freund adjuvant and the remaining doses were with incomplete Freund adjuvant at 15-day intervals. Two weeks after the last dose, animals were bled and the sera were stored at −20°C.

#### Enzymatic assays

Epimastigotes and trypomastigotes of *T. cruzi* (10^8^ cells) were harvested and washed in PBS. The parasites were resuspended in 1 mL of lysis buffer (80 mM HEPES pH 7.5/150 mM NaCl/1 mM MgCl_2_/1% NP 40/3 mM EGTA/3 µL DNase) during 10 min in an ice-bath. The solutions were centrifuged (10,000 x *g*; 10 min; 4°C) and the soluble (S) and insoluble (P) fractions were separated. The P fractions were washed twice and resuspended in 1 mL of the same buffer. Calpain activity of freshly prepared S and P fractions was assayed by both gel enzymography and fluorometry. For this, 5 or 10 µL of each fraction were submitted to 8% SDS-PAGE-0.2% gelatin at 4°C under reducing or non-reducing conditions, without boil the samples. After running, the gel was washed 4 times with 25 mM Tris-HCl/150 mM NaCl, pH 7.5 (reaction buffer), and incubated in the same buffer containing 0.25, 1.0 or 2.0 mM CaCl_2_, at 37°C, during 24 h. The gel was then Coomassie stained. Calpain activity was also assayed by diluting 10 µL of S or P fractions in 90 µL of reaction buffer containing different concentrations of CaCl_2_ (from 0.062 to 2.0 mM) in the presence of 25 mM N-Suc-Leu-Leu-Val-Tyr-7-amido-4-Methilcoumarin (LLVT-AMC; Sigma-Aldrich), a substrate of the CA clan of cysteine peptidases (www.merops.sanger.ac.uk). After 30 min incubation, free AMC was measured in a HITACHI F-2000 spectrofluorometer as described [34].

#### SDS-PAGE and Western blotting

*T. cruzi* epimastigotes (2×10^6^ cells) were harvested by centrifugation, washed in PBS for 5 min at 1,600 x *g* and lysed with a solution containing 150 mM NaCl/80 mM PIPES (pH 7.2)/1 mM MgCl_2_/3 mM EGTA/3 mM EDTA/1% Triton X-100/0.1 mM AEBSF/0.5 mM 1,10-phenanthroline/2.2 µM Pepstatin/1.4 µM E-64. The parasite lysate was incubated at 4°C for 10 min and centrifuged for 5 min at 17,400 x *g* at 4°C. These supernatants were used for the immunoblotting studies. Pellets and supernatants were boiled in Laemmli's sample buffer and subjected to SDS-PAGE (5%) using molecular size markers ranging from 205 kDa to 29 kDa (Sigma SDS-6H) as a reference. Western blots were performed using standard procedures [35]. The western blots were probed with the appropriate anti-sera (against H49 and CysPc domain) and after reaction with anti-mouse IgG conjugated to horseradish peroxidase, were developed by chemiluminescence using the ECL Western blotting detection reagent and Hyperfilm (Amersham Biosciences).

### Immunolocalization

#### Immunofluorescence localization in whole cells

*T. cruzi* epimastigotes were centrifuged and washed with PBS for 5 min at 900 x *g*. Parasites were fixed with 2% paraformaldehyde diluted with PBS and incubated for 30 min at room temperature. The cells were washed three times with PBS for 5 min at 900 x *g*. The fixed parasites were placed on glass slides. The parasites were permeabilized with 0.1% Triton X-100 for 5 min at room temperature, washed and incubated sequentially with anti-H49, anti-CysPc or anti-H49 deg at an appropriate dilution for one hour at room temperature. The slides were washed and incubated for one hour with an appropriate dilution of fluorescein (FITC)-conjugated anti-mouse (IgG) or anti-rabbit IgG diluted in PBS/10% serum containing 10 µM DAPI.

#### Immunofluorescence localization in cytoskeleton

Epimastigote forms were placed on glass slides in a humid chamber for 30 min. Decanted parasites were briefly washed with PBS. To prepare the cytoskeleton, cells were lysed on glass slides with a solution containing 150 mM NaCl/80 mM PIPES pH 7.2/1 mM MgCl_2_/3 mM EGTA/3 mM EDTA/0.5% NP-40 or 1% Triton X-100/0.1 mM AEBSF/0.5 mM 1,10-phenanthroline/2.2 µM Pepstatin/1.4 µM E-64. The cytoskeletons were fixed with 2% paraformaldehyde at room temperature for one hour, washed with PBS and stored refrigerated in a humid chamber until use. The cytoskeletons were incubated with anti-H49, anti-CysPc or anti-H49 deg, and DAPI and FITC-conjugated secondary antibody as described above. Images were acquired on a Nikon E600 fluorescence microscope coupled to a Nikon DXM 1200F digital camera using ACT-1 software. Images were processed with Image J and Adobe Photoshop 7.0.

#### Cloning of various regions of H49/calpain genes by reverse transcriptase PCR

Total RNA was extracted from epimastigotes with TRIzol®. First-strand cDNA was prepared using the ThermoScript™ RT-PCR System (Invitrogen) according to the manufacturer's instructions. Specific primers based on sequences of H49/calpains XM_799896, XM_804900 and XM_799016 were used to amplify CysPc, H49 deg and H49 conserved, respectively (Table S2). In addition, these primers were used to amplify H49/calpain sequences in genomic DNA of *T. cruzi*. The amplified PCR products were cloned into plasmid pGEM®-T easy vector (Promega) and transformed into *E. coli* strain DH-5α. Nucleotide sequences of cDNA clones and genomic DNA clones were determined using the dideoxynucleotide chain termination method with BigDye Terminator cycle sequencing chemistry (Applied Biosystems) in an ABI PRISM 377 DNA Sequencer.

#### Sequence similarity searches

The *T. cruzi* clone CL Brener [27] genome sequence used in this study was obtained from the National Center for Biotechnology Information (http://www.ncbi.nlm.nih.gov/GenBank). A locally compiled database (DB) of *T. cruzi* sequences was built by parsing sequences from GenBank, GeneDB (http://www.genedb.org), and The Institute for Genomic Research, and used for sequence similarity searches. Similarity searches of amino acid and nucleotide sequences of H49 repeats (GenBank L09564) against this locally compiled DB were carried out using the BLAST and FASTA program package algorithms [36]. To search for, identify and extract H49 repeat full copies in this locally compiled sequence DB, we used a PERL script specifically developed for this study and loaded with a regular expression specifically describing the repeat. The annotation and graphical output of the H49 repeats and flanking regions were obtained using ARTEMIS [37] (http://www.sanger.ac.uk/Software/Artemis) and in-house-developed PERL scripts to analyze and format the results of the similarity searches. The alignments were realized in January 2009 using BLAST 2.2.19 version and only alignments with an E-value between 0 and 1×10^−3^ were chosen. In addition, a search in the GenBank database, using the key words calpain OR calpain-like AND *T. cruzi* CL Brener, was carried out to identify calpain sequences.

#### Global multiple sequence alignments of domains II (CysPc) and III of calpains identified in the *T. cruzi* database and phylogenetic inference

Global multiple sequence alignments of domains II (CysPc) and III of calpains identified in the *T. cruzi* database were performed using Clustal X [38] followed by visual inspection and manual adjustment with SeaView [39] (http://pbil.univ-lyon1.fr/software/seaview.html) and GeneDoc (http://www.psc.edu/biomed/genedoc). The phylogenetic analysis was performed using MEGA 4 program [40]. The phylogenetic tree was obtained for the Neighbor-joining method and constructed using an input model with 5,000 bootstrap replications.

## Results and Discussion

### Comparative sequence analysis to identify genes carrying H49 repeats in the *T. cruzi* genome

The clone H49, isolated by immunoscreening from a *T. cruzi* expression library, consists of 4.8 tandemly arranged repeats of 204-bp that encode 68-amino acid repeats located in a high molecular weight cytoskeleton-associated protein [17], [23].

**Figure 1:**
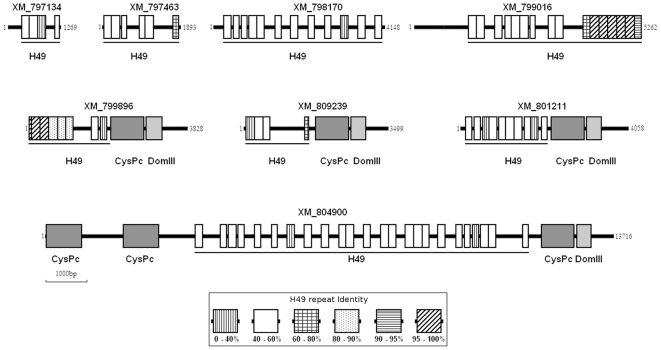
Schematic representation of H49/calpain genes identified in the *T. cruzi* genome by tblastn using the 204-bp repeat of clone H49 (accession no. L09564) as the query. The repeats are boxed and their identity to the H49 repeat (query) is indicated at the foot of the figure. The specific calpain domains CysPc and III are boxed and shaded in dark and light gray, respectively. Accession numbers for nucleotides and translated protein (in parentheses) sequences are: XM_797134 (XP_802227), XM_797463 (XP_802556), XM_798170 (XP_803263), XM_799016 (XP_804109), XM_799896 (XP_804989), XM_809239 (XP_814332), XM_801211 (XP_806304) and XM_804900 (XP_809993).

The sequence of clone H49 (GenBank L09564) was used as a query to search for H49 genes in the *T*. *cruzi* genome database (GeneDB and GenBank) using the tblastn program. Twenty-three contigs consisting solely of conserved tandem repeats of 204-bp, and eight contigs carrying the 204-bp repeats associated with the calpain-like cysteine peptidase sequences (Figure 1), were identified, and are referred to as H49/calpains herein. These proteins possess the CysPc calpain domains IIa and IIb, characteristic of calcium-dependent cytoplasmic cysteine proteinases and papain-like proteins (Figure 1 and Table S1). Among the 53 *T. cruzi* calpain-like sequences in the genome database, eight were associated with H49 sequences. Only one H49/calpain sequence (XM_804900) represents an entire gene copy; the remaining copies were incomplete and collapsed in the repeat region. Collapsed repeats frequently arise during automated genome assembly when sequence reads originating from distinct repeat copies cannot be joined to generate a single unit. Two H49/calpain pseudogenes (Tc00. 1047053506925.550 and Tc00.1047053511443.10) were also identified.

The blocks of tandemly arranged, conserved 204-bp (68-aa) repeats are flanked by degenerate repeats present in various numbers (Figure 1). Herein, H49 conserved and H49 degenerate denote units that are more or less than 80% similar to the H49 unit (GenBank L09564) using tblastn analysis, respectively. H49 conserved units were located at the extremities of the following sequences: XM_799016, XM_799896 and XM_797463 (Figure 1); H49 degenerate units were identified in all H49/calpains. For example, the XM_799016 sequence (Figure 1) consists of inexact repeats separated by short non-repeat sequences, followed by a block of tandemly conserved 204-bp repeats at the 3′ end. The only entire copy of the H49/calpain gene (XM_804900) found in the *T. cruzi* genome database encodes a protein of 4,571 amino acids (∼520 kDa), which contains 24 degenerate repeats in its central domain (Figure 1).

Blastp search revealed the presence of inexact H49 repeats in genomes of other trypanosomatids including *Trypanosoma brucei, Trypanosoma brucei gambiense, Leishmania major, Leishmania infantum* and *Leishmania braziliensis*. These repeats are less than 60% identical to H49, and are located in calpain-like cysteine peptidases in *Leishmania spp.* and in calpain-related proteins (CAP5.5) in *T. brucei* [41], [42]. These results suggest that the expansion of H49 repeats only occurred in *T. cruzi* after separation of a *T. cruzi* ancestor from other trypanosomatid lineages. The expansion could be generated by a recombination process that homogenizes tandemly repeated sequences, for instance unequal crossing over after misalignment of repeats and/or gene conversion. The H49/calpain gene (XM_804900), which encodes the calpain (XP_809993) carrying degenerate H49 repeats, could be a remnant of ancestral repeats that rarely participated in the recombination processes that maintain the present tandem repeat arrays.

The H49/calpains can be classified as calpain-like proteins (CALP). They carry one copy of the protease domain [domain II (CysPc) followed by domain III] in the carboxy-terminal region, and two copies of CysPc in the amino-terminal region. Between the protease domains is a region composed of tandem repeats of 65–68 amino acid residues unit length. Four H49/calpains proteins (XP_806304, XP_814332, XP_804989, XP_809993) contain the domains II (CysPc) and III. The H49/calpains XP_806304 (1,351 aa) and XP_814332 (1,165 aa) share 72% identity, and 52–54% with XP_804989 (1,275 aa). Comparison with the protein XP_809993 (4,571 aa) is somewhat complicated by its great length. The amino acid sequence identity between XP_809993 and XP_814332 over the first 1,165 amino acids is 98%, and 75–77% with XP_806304 and XP_802263. Domains II (CysPc) and III of H49/calpains were compared with other *T. cruzi* calpain-like cysteine peptidases and calpain cysteine peptidases (Figs. S2 and S3). The CysPc and III domains are conserved among H49/calpains (Figure S4) but they differ from other *T. cruzi* calpains. Residues of the classic CHN (Cysteine-Histidine-Asparagine) cysteine protease catalytic triad were partially conserved in H49/calpains, suggesting that these proteins do not mediate peptidase activities. This correlates well with our observations that the insoluble *T. cruzi* fractions do not mediate calpain activity in contrast with the soluble fractions that hydrolyzed LLVT-AMC substrate (data not shown).

**Figure 2:**
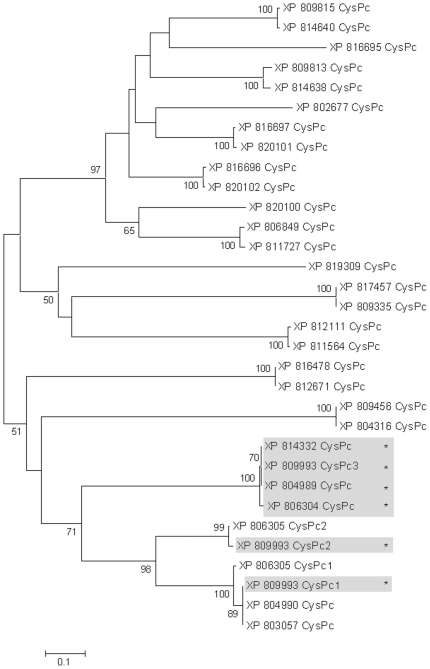
Phylogenetic reconstruction of the CysPc domain of *T. cruzi* calpain cysteine peptidases classified as calpain and calpain-like cysteine peptidases. H49/calpains are shaded and indicated by an asterisk (*). Sequences XP_809993 and XP_806305 have three and two CysPc domains, respectively, which are indicated by numbers 1, 2 and 3. Bootstrap values not shown are below 50%.

A phylogenetic tree based on the CysPc of *T. cruzi* calpain-like proteins is presented in Figure 2. The CysPc domains of H49/calpain are grouped into a cluster (bootstrap 100%), separated from the other *T. cruzi* calpain-like proteins. Interestingly, one CysPc domain of H49/calpain XP_809993 is part of one cluster, while two other CysPc domains present in this protein have similarities with other calpains. The phylogenetic reconstruction suggests that the CysPc domains of H49/calpain are derived from a common sequence (Figure 2). Interestingly, this group includes three *T. cruzi* calpain-like proteins (XP_806305, XP_804990 and XP_803057) that do not contain the 68-aa repeats. The sequences coding for XP_806305 and XP_803057 are incomplete, interrupted by errors during DNA sequencing. They share more than 98% identity with the amino-terminal region of XP_809993, suggesting that they could be paralogs of XP_809993, a H49/calpain that carries degenerate H49 repeats (see Figure 1). Recently, Giese *et al*. identified a *T. cruzi* calpain-like peptidase (XP_816697) expressed in epimastigotes subjected to nutritional stress that precedes metacyclogenesis [43]. The CysPc domain of this protein is grouped with other calpain-like proteins that do not contain 68-aa repeats.

### Genomic organization of H49/calpain genes

The H49/calpain genes are distributed among 10 contigs (ranging in length from 1,269 to 36,049 bp) assigned to the chromosome-sized scaffolds TcChr39-P and TcChr39-S of the *T. cruzi* sequenced genome (clone CL Brener). Recently, *T. cruzi* contigs were assembled into 41 chromosome-sized scaffolds named chromosomes (TcChr), which were numbered in crescent order size [44]. Clone CL Brener is a hybrid that displays two haplotypes. Therefore, the chromosome-sized scaffolds assigned to the Esmeraldo and non-Esmeraldo haplotypes were designated S and P, respectively. All H49/calpain genes were densely clustered within a distance of ∼45 Kb on the genome, one cluster composed of three genes (XM_804900, XM_799016 and XM_798170) on TcChr39-P, another composed of five genes and two pseudogenes on TcChr39-S (Figure S1). The functional H49/calpain genes XM_804900 and XM_798170 are located on TcChr39-P, and their pseudogenes are located on TcChr39-S. Each of the H49/calpain genes in a cluster is in the same transcription orientation.

**Figure 3:**
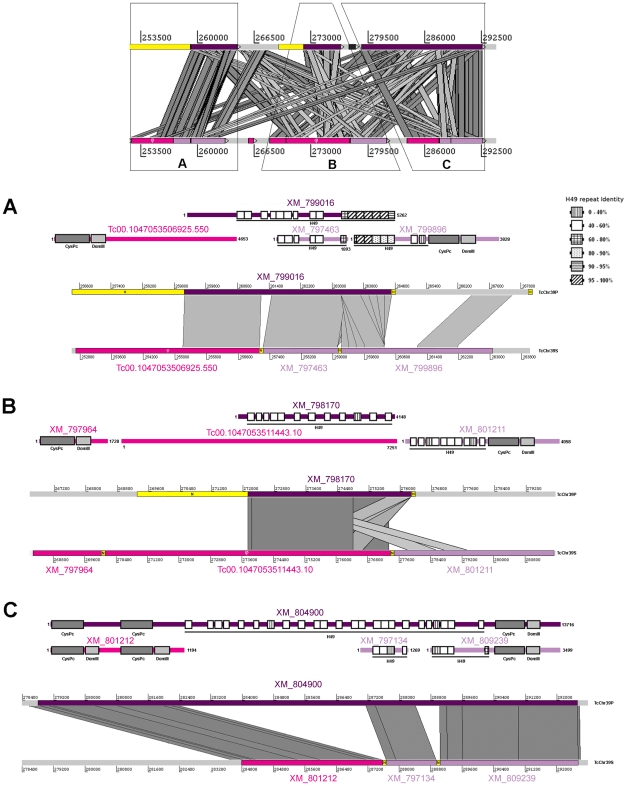
Schematic overview of the genomic region containing the H49/calpain genes on the *T. cruzi* chromosomes TcChr39-P and -S. “S” chromosome is assigned to the Esmeraldo haplotype and “P” to the non-Esmeraldo haplotype. Each delimited area is presented in zoom-in panels A, B and C. **Panel A**) Detailed representation of region A demonstrating overlap of genes H49/calpain XM_799016 belonging to TcChr39-P, calpain-like pseudogene Tc00.1047053506925.550 and H49/calpains XM_797463 and XM_799896 belonging to TcChr39-S. **Panel B**) Alignment among homologous regions carrying H49/calpain gene XM_798170 (TcChr39-P) and XM_797964 (calpain-like), and pseudogene Tc00.1047053511443.10 and H49/calpain XM_801211 belonging to TcChr39-S. **Panel C**) Detailed representation of region C demonstrating alignment among H49/calpain XM_804900 (TcChr39-P) and calpain gene XM_801212 and H49/calpain genes XM_797134 and XM_809239. Calpain/H49 genes belonging to TcChr39-P and -S are represented by dark and light purple rectangles, respectively. Sequences deposited in the *T. cruzi* database as calpain-like are indicated by pink rectangles. Homologous regions are connected by gray lines. N regions (nucleotide not determined) present in both TcChr are indicated by yellow blocks marked by the letter N. Above each panel is the schematic representation of each sequence located in the specific alignment region between TcChr39-P and -S. The length (in bp) is indicated to the right of each gene. The symbol ϕ indicates a pseudogene. The repeats are boxed and their identity to the H49 repeat is indicated at right of the figure. The specific calpain domains CysPc and III are represented by dark and light gray rectangles, respectively.

There are several contigs carrying repetitive sequences at one or both ends, undetermined regions of nucleotides (hereafter called N regions) were introduced between two contigs. Figure 1 presents seven H49/calpains containing tandem repeats at one or both extremities. Interestingly, all gene-ends contain H49 repeats flanked by N regions. There is overwhelming evidence that these repetitive sequences prevent the correct assembly of the complete H49/calpain genes. The only complete H49/calpain sequence identified in the *T. cruzi* database is XM_804900, located on the chromosome-sized scaffold TcChr39-P (Figure S1). Flanking H49 degenerate units are two CysPc domains (A, B) in the 5′ region, a third (C) and a domain III in the 3′ region (Figures S1 and S4). The alignments between TcChr39-S and -P chromosomes revealed the relationships among H49/calpains. Figure 3 presents the overall gene-to-gene comparison between TcChr39-S and -P chromosomes, and highlights a set of calpain genes in more detail. The comparison demonstrated similarity between the 5′ ends of calpains XM_804900 and the XM_801212, and between the 3′ ends of XM_804900, XM_809239 and XM_797134 (Figure 3C). XM_801212 has two blocks consisting of CysPc and domain III. XM_797134 contains four H49 repeats, whereas XM_809239 contains H49 repeats followed by CysPc and domain III. XM_801212, XM_797134 and XM_809239 are separated by N regions, suggesting a collapsed problem in TcChr39-S. Phylogenetic analysis of the CysPc domain (Figure 2 and Figure S4) demonstrated that the three CysPc (A, B, C) domains of XM_804900 share high sequence identity with the corresponding CysPc domains in XM_801212 (CysPc A and B) and XM_809239 (CysPc C). The location in homologous regions, the collapsed problem indicated by N region and the strong similarity among CysPc domains suggest that these sequences (XM_801212, XM_797134 and XM_809239) could be parts of the same gene, a homologue of XM_804900.

Chromosomal alignment was used to define the relationship among other H49/calpain sequences located on chromosomes TcChr39-P and TcChr39-S (Figure 3A and B). Various regions of XM_799016, located on TcChr39-P, share similarity with three sequences located on TcChr39-S: a calpain-like pseudogene Tc00.1047053506925.550, and H49/calpains XM_797463 and XM_799896 (Figure 3A). All of these sequences are flanked by N regions. Chromosomal alignment suggests that Tc00.1047053506925.550, XM_797463 and XM_799896 belong to a H49/calpain pseudogene located on TcChr39-S. The same reasoning suggests that a calpain-like cysteine peptidase pseudogene (Tc00.1047053511443.10) and H49/calpains XM_797964 and XM_801211 could be parts of a H49/calpain pseudogene.

Similarity between H49/calpain sequences on TcChr39-P and TcChr39-S scaffolds was greater than similarities between adjacent H49/calpain genes located within the same scaffold. We suggest that there are six H49/calpain sequences in the genome of clone CL Brener: two truncated copies (XM_799016 and XM_798170) and one complete copy (XM_804900) on TcChr39-P; two truncated copies XM_797463 and XM_799896) and a pseudogene (Tc00.1047053506925.550) on TcChr39-S. It is noteworthy that CysPc domains share high sequence identity according to their position in the molecule (Figure S2). Calpain genes that do not contain H49 repeats are distant phylogenetically and have completely different amino acid sequences from the H49/calpain proteins.

Chromosomal bands were separated by PFGE and hybridized with various regions of H49/calpain genes (H49 repeat, catalytic domain CysPc and H49 degenerate repeats) as probes. Previously, H49 repeat has been mapped to the chromosomal bands XVI and XVII of clone CL Brener, which are homologous chromosomes of different sizes [45]. As expected, probes from CysPc, and H49 conserved and degenerate repeats hybridized to the same chromosomal bands (data not shown). The physical link between H49 repeats and CysPc domain was confirmed by genomic Southern blot hybridization (data not shown).

### Expression and cellular location of H49/calpain

To confirm the transcription of H49/calpain genes in epimastigotes, a series of RT-PCR amplifications was performed with sets of primers covering the H49 repeat and CysPc coding sequences of H49/calpain genes (Figure S5). PCR products with expected sizes were cloned and their identity confirmed using sequencing. These results indicate that H49/calpain genes are transcribed in epimastigotes.

To identify the H49/calpain proteins and their cellular location, antibodies were raised against various recombinant proteins carrying the catalytic CysPc domain (XP_804989), the conserved 68-aa repeats found in clone H49 (L09564) and the degenerate repeats and calpain sequence present in XP_809993. Anti-sera to CysPc domain, 68-aa repeats and H49 degenerate repeats were named anti-CysPc, anti-H49 and anti-H49 deg, respectively. The degenerate repeats share less than 80% identity with the conserved H49 repeats and this could explain the weak cross-reaction between these antigens detected by dot blots and western blotting.

**Figure 4:**
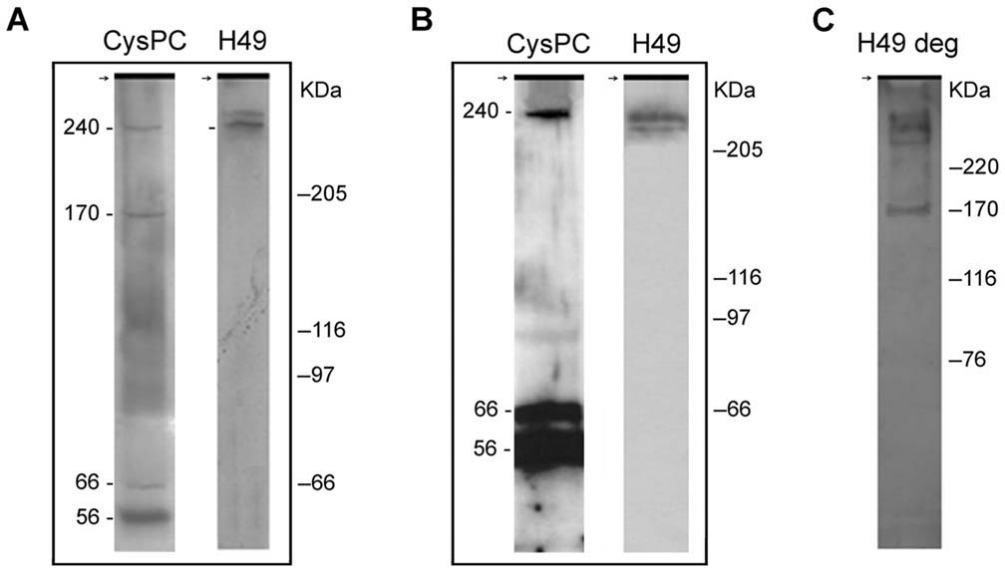
Western blotting of cytoskeletal fractions of epimastigotes of clone CL Brener (A) and G strain (B–C) with rabbit polyclonal antibodies against the catalytic domain of calpain (CysPc), H49 conserved and degenerate (H49 deg) repeats. The cytoskeletal fraction of Triton X-100 extracted cells was resuspended in denaturing sample buffer, and proteins were separated on 5% SDS-PAGE. Nitrocellulose membranes were incubated with the antibodies as indicated in the Methods sections. The molecular sizes of immunoreactive proteins and standard molecular weight markers are indicated on the left and right, respectively.

In order to investigate the possible protein–membrane association of H49/calpains, epimastigotes were lysed with the non-ionic detergent Triton X-100, which solubilizes membrane proteins. Proteins from the cytoskeleton fraction were separated by SDS-PAGE and reacted with antibodies. On western blots, antibodies against the conserved repeats reacted with double bands of approximately 240 kDa in clone CL Brener and G strain (Figure 4). Anti-CysPc antibodies reacted with one member of the 240 kDa doublet, with three additional bands in clone CL Brener (56, 66 and 170 kDa) and two bands in G strain (56 and 66 kDa) that correspond to calpain-like proteins without repeats. The fact that CysPc antibodies detected only one band of the 240 kDa doublet, rather than the two bands observed with anti-H49 antibodies, suggests the presence of a H49/calpain variant without the CysPc domain.

Antibodies against the degenerate repeats reacted with three proteins in the 240 kDa range and with a 170 kDa protein (Figure 4). Calpain-like CAP5.5 and flagellar calcium binding protein are linked to the plasma membrane via palmitic acid and/or myristic residues [41], [46]. However, acylation sites were not identified at the amino-terminal domain of H49/calpains, suggesting that they are not linked to cellular membranes.

**Figure 5:**
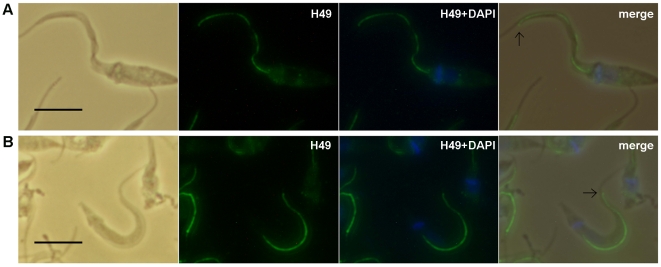
H49 repeats are located along the FAZ region in whole parasite cells. Epimastigotes (**A**) and trypomastigotes (**B**) from G strain were fixed with paraformaldehyde (2%), permeabilized with Triton X-100 (0.1%), and incubated with anti-H49 antibodies. Immunocomplexes were detected with anti-mouse IgG-Alexa 488 (second panel in green). In third panels are shown the merge of anti-H49 (green) and DAPI (blue). At left, panels show the corresponding phase-contrast image and at right, merged image of the two fluorescent channels and phase-contrasting. The arrows (at right panels) indicate the end of the FAZ region. Bar, 5 µm.

To identify the cellular locations of calpain-like proteins in *T. cruzi* using immunofluorescence microscopy, the same anti-sera used for the biochemical analysis were employed. Antibodies against the 68-aa repeats produced a clearly defined intense staining pattern, located exclusively in the FAZ (Flagellar Attachment Zone) (Figure 5). Immunolocalization revealed that antibodies against H49 repeats reacted in the anterior region of the parasite, where the flagellum becomes free (Figure 5).

**Figure 6:**
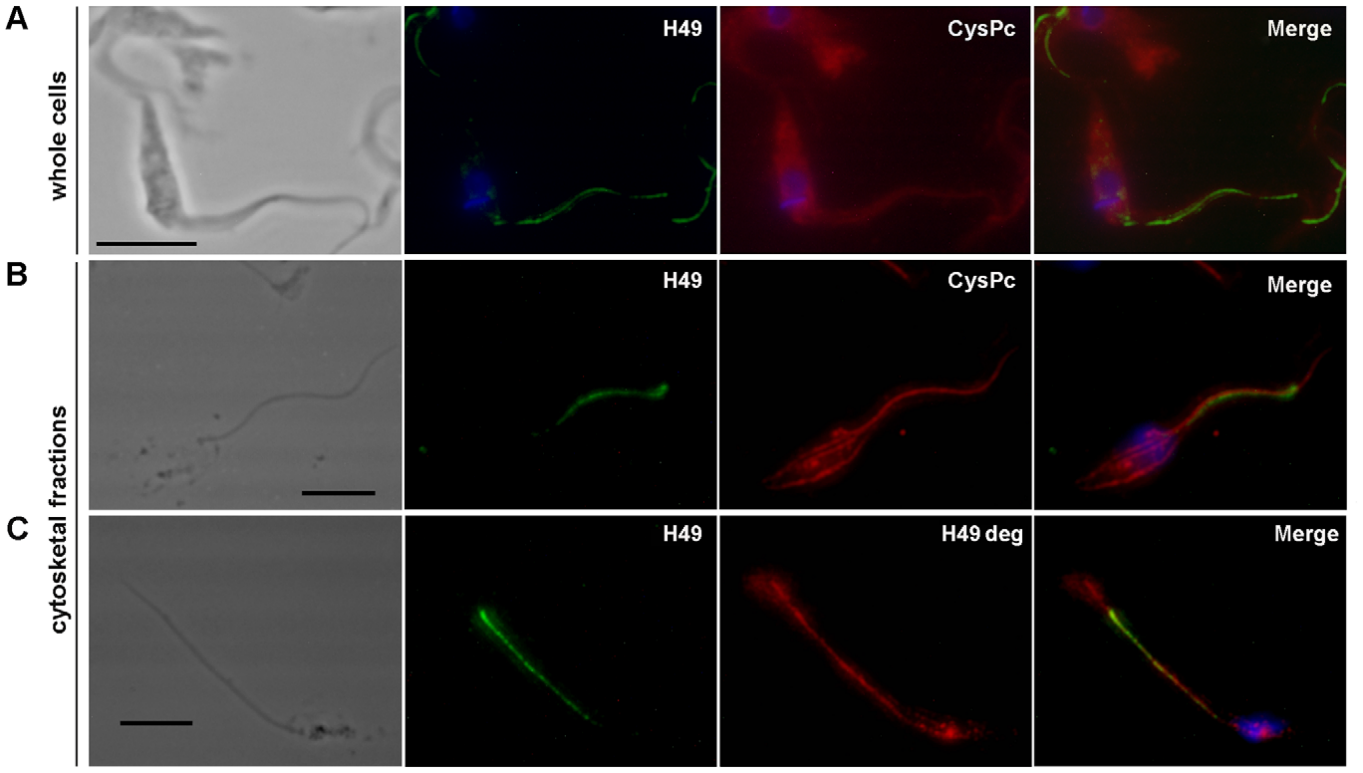
H49 conserved repeats (H49) co-localize with CysPc domain (CysPc) and with degenerate repeats (H49 deg) in whole parasite cells and in cytoskeletal fractions. **A**. Epimastigotes (CL Brener) were fixed with 2% paraformaldehyde, permeabilized with Triton X-100 (0.1%) and incubated with mouse anti-H49 and rabbit anti-CysPc antibodies. Primary antibodies were recognized using anti-mouse IgG-FITC secondary antibodies (second panel, in green) and anti-rabbit IgG-Texas Red secondary antibodies (third panel, in red). **B and C**. Cytoskeleton fractions of epimastigotes (CL Brener) were obtained by lysis with Nonidet P40 (0.5%) and fixed with 2% paraformaldehyde. **B**. Fractions were incubated with mouse anti-H49 and rabbit anti-CysPc antibodies. Primary antibodies were recognized using anti-mouse IgG-FITC secondary antibodies (second panel, in green) and anti-rabbit IgG-Texas Red secondary antibodies (third panel, in red). **C**. Samples were revealed with rabbit anti-H49 and mouse anti-H49 deg antibodies followed by anti-rabbit IgG-Alexa 488 (second panel, in green) and anti-mouse IgG-Alexa 564 (third panel, in red) secondary antibodies. DNA was stained with DAPI (blue). At left, panels show the corresponding phase-contrast image and at right, merged image of the three fluorescence channels. Bar, 5 µm.

Figure 6A presents the co-localization assays concerning the H49 repeats and the CysPc domain in parasites permeabilized with Triton X-100. The anti-CysPc antibodies reacted with components of the cytoplasm and along the entire flagellum, whereas anti-H49 antibodies stained the FAZ region of the parasite. The epitopes recognized by anti-CysPc and anti-H49 antibodies appear to be very close, and they co-localize at several points. This result is in agreement with the western blotting results demonstrating the presence of H49/calpains that do not contain the CysPc domain.

To investigate the cellular distribution of H49/calpain further, epimastigotes were lysed with non-ionic detergent (Nonidet-40 or Triton X-100) and the resulting cytoskeletons were analyzed using immunofluorescence. Anti-H49 antibodies reacted with the cytoskeleton producing a punctate pattern exclusively in the region of the FAZ (Figure 6B). In contrast, anti-CysPc reacted with the subpellicular cytoskeletal microtubules and along the length of the flagellum. H49 repeats and CysPc domain were co-localized at several points in the FAZ region (Figure 6B). The staining pattern of anti-H49 deg antibodies was similar to that observed with anti-CysPc antibodies in cytoskeleton preparations of epimastigotes lysed with NP-40 (0.5%) (Figure 6C and Figure S6). Previously, we characterized a monoclonal antibody, 4D9, which reacted with a high molecular weight component located in the cell body of the FAZ region [10], [24], [25]. In this study, the distribution of H49/calpains was compared with the 4D9-reactive antigen in cytoskeletal fractions (Figure S7). The monoclonal antibody 4D9 does not cross-react with H49/calpains, indicating that it recognizes another molecular entity. Furthermore, 4D9-reactive antigen is located in the FAZ region, as previously reported [10], [24]. The labeling pattern obtained using 4D9 was the same as that obtained with anti-H49 antibodies, both antigens being located at the FAZ region (Figure S7). The calpain component is distributed throughout the cytoplasm and along the flagellum (Figure S7).

Immunofluorescence analysis with anti-H49 antibodies demonstrated that H49 conserved repeats were not detected in the anterior end of the flagellum but were restricted to the FAZ region. It is likely that the H49 antigen is a component of the FAZ in *T. cruzi*, similar to the protein FAZ1 in *T. brucei*. However, antibodies against the H49 degenerate repeats present on the H49/calpain (XP_809993) reacted with the cytoskeleton microtubules and along the flagellum including the anterior end and FAZ regions. Therefore, the *T. cruzi* H49/calpain carrying degenerate repeats (XP_809993) is related to the calpain-like protein CAP5.5 of *T. brucei*. These differences suggest that phylogenetically related H49/calpains can have different functions in the parasite.

The catalytic activity of classical calpains is determined by the presence of the catalytic triad (CHN) present in domain II (CysPc) and the EF-hand motifs that bind calcium, present in domain IV [47]. The calpains that differ from this configuration are classified as "calpain-like" (CALP) [48]. The H49/calpains can be classified as calpain-like (CALP), and two of them (XP_804109 and XP_804989) can be annotated as putative calpain cysteine peptidase. As demonstrated using immunofluorescence analysis, the H49/calpains are located in the FAZ region of the parasite. The proteins in the FAZ region of *T. brucei* have high molecular weights and are expressed at low levels, hindering detection [49]. In *T. brucei*, the FAZ1 protein is associated with the cytoplasmic FAZ filament [16]. This protein contains 14-aa repeats and migrates in SDS-PAGE as high molecular weight bands (>200 kDa) similar to H49/calpains.

Immunoblotting demonstrated that H49/calpains are retained in the cytoskeletal fraction of clone CL Brener and G strain epimastigotes. Moreover, anti-sera against the repeats and CysPc domain reacted with components of the soluble fraction. Recently, Giese *et al*. (2008) demonstrated the presence of a calpain-like protein in the detergent-soluble and insoluble fractions of *T. cruzi* [43]. This could be due to the translocation of cytoplasmic calpains to the plasma membrane after cellular stimulation. Ennes-Vidal et al. (2011) showed by ultrastructural immunolabeling that calcium-dependent cysteine peptidases are mainly located at the cytoplasm of epimastigotes [50].Recently, we identified seven calpain-like proteins including two cytoskeletal associated proteins CAP5.5 in the plasma membrane of *T. cruzi* epimastigotes and metacyclic trypomastigotes [51]. Six of these calpain-like proteins contain N-terminal fatty acid acylation motifs, indicating an association with cellular membranes.

### Function of H49/calpains

As *T. brucei* calpain related proteins, CAP5.5 and CAP5.5V, H49/calpains lack the C-H-N catalytic triad, suggesting that they do not have catalytic activity. Preliminary results using a biochemical assay indicated that H49/calpains do not have proteolytic activity *in-vitro* (data not shown). Several authors have been suggested that loss of proteolytic capacity would have been an early step in the evolution of calpain like-proteins in mammals and trypanosomes as a microtubule-interacting proteins [42], [50].The H49/calpain is located in the FAZ region and remains tightly associated with the cytoskeleton after the extraction of cellular membranes with non-ionic detergents. On the cytoplasmic side of the FAZ there is an electron-dense filament positioned adjacent to a specialized group of four endoplasmic reticulum-associated microtubules that run from the flagellar pocket to the anterior end of the cell [7], [13], [52]. The FAZ filament is connected to the flagellum by a network of filaments [53]. The flagellum-cell body attachment is due to a tight physical connection between the cytoplasmic filament of the FAZ and the flagellar filament linked to the proximal domain of the PFR [7].

Recent results from our laboratory (Mortara, RA, unpublished results) have demonstrated that the 4D9-reactive antigen is located in a region between the flagellum axoneme and the subpellicular microtubules. Immunolabeling of *T. cruzi* cytoskeletons using transmission electron microscopy demonstrated that labeling was associated with filamentous elements in juxtaposition with subpellicular microtubules, suggesting a possible association of 4D9-reactive antigen with the FAZ region. By analogy, it is suggested that H49/calpain could have a structural role in maintaining attachment of the flagellum to the cell body, connecting the subpellicular microtubule system to the flagellum. The H49/calpain could be associated with the FAZ cytoplasmic filament, which interacts with connecting filaments and the subpellicular microtubules.

Bisaggio *et al*. analyzed the effects of suramin, which affect the synthesis and distribution of cytoskeletal proteins, on *T. cruzi* trypomastigotes [24]. This inhibitor caused a significant increase in the phenotypic expression and distribution of H49 antigen in the region of adhesion between the cell body and the flagellum of trypomastigotes. Morphological alterations in terms of flagellar attachment were observed after suramin treatment in *T. cruzi* and were similar to the effects caused by blocking trypanin expression in *T. brucei*, and it is suggested that suramin could inhibit a *T. cruzi* protein similar to *T. brucei* trypanin.

## Supporting Information

Figure S1 **Schematic overview of the genomic regions containing the H49/calpain genes on the** ***T. cruzi*** **chromosome sized scaffolds TcChr39-P and -S.** Arrangement of the H49/calpain gene family in chromosomal clusters TcChr39-P and -S (“S” chromosome assigned to the Esmeraldo haplotype and “P” to the non-Esmeraldo haplotype). Comparison between regions from TcChr39-P and -S containing H49/calpains. Homologous genes are connected by gray lines. H49/calpain genes belonging to TcChr39-P and -S are represented by dark and light purple rectangles, respectively. Sequences deposited in the *T. cruzi* database as calpain-like are indicated by pink rectangles. Locus names are written below each rectangle. The symbol ϕ indicates a pseudogene. Green rectangles represent hypothetical proteins and numbers 1, 2 and 3 inside blue rectangles correspond to RNA processing factor 1, ABC transporter and radial spoke protein 3, respectively. N regions (nucleotide not determined) are indicated by yellow blocks marked by the letter N. Above and below the alignment are regions of TcChr39-P and TcChr39-S, respectively. Genes are drawn in sense strand (signals +) and antisense strand (signal -).
(TIF)

Figure S2 **Multiple alignment of the CysPc domain of** ***T. cruzi*** **calpain-like proteins.** Identical residues are highlighted in black; dark gray, 80% identity; and light gray, 60% identity. The accession numbers of amino acid sequences are indicated on the right, omitting the initial letters XP. Sequences appear in the same order as they appeared in the phylogenetic tree (Figure 2). In this figure, the CysPc domain of the pseudogene Tc00.1047053506925.550 was included and is indicated by Tc00CysPc. Sequences XP_809993 and XP_806305 have three and two CysPc domains, respectively, which are indicated by numbers 1, 2 and 3.
(TIF)

Figure S3 **Multiple alignment of the domain III of** ***T. cruzi*** **calpain-like proteins.** Identical residues are highlighted in black; dark gray, 80% identity; and light gray, 60% identity. The accession numbers of amino acid sequences are indicated on the right, omitting the initial letters XP.
(TIF)

Figure S4 **Alignment of the first, second and third domains CysPc of H49/calpains.** CysPc domains of H49/calpains were grouped in different arms of the phylogenetic tree (Figure 2). According to the position in chromosomes TcChr39-P and -S, the complete sequences of these calpains were predicted (Figure 4). The first (A), second (B) and third (C) CysPc domains were aligned using the ClustalW method. At letter C, the third CysPc domains are boxed in red and domain III are boxed in blue. Identical residues are highlighted in black; dark gray, 80% identity; and light gray, 60% identity. The accession numbers of amino acid sequences are indicated on the right, omitting the initial letters XP. Sequences XP_809993 and XP_806305 have three and two CysPc domains, respectively, which are indicated by numbers 1, 2 and 3.
(TIF)

Figure S5 **RT-PCR amplification of H49/calpain sequences from epimastigotes (CL Brener).** Left) The primers were based on the sequence of H49 repeats and catalytic domain (CysPc) from H49/calpains indicated in the figure. The regions A and B containing the degenerate H49 repeat of gene XM_804900 (XP_809993) were amplified using the primers Calp520(1) and H49deg (1) and H49deg(2) and Calp520(2), respectively. The regions C and D from gene XM_799896 (XP_804989), containing the catalytic domain of calpain (CysPc) and the CysPc and H49, were amplified using the primers Tc(1) and Tc(2), and H49(3) and Tc10(2). The region D from gene XM_799016 (XP_804109) was amplified using the primers Tc(3) and H49(2). Right) Electrophoresis on agarose gels demonstrating the amplicons carrying the various regions (A–E) of H49/calpain genes. The control was carried out with total epimastigote RNA treated with DNase. The molecular size markers were indicated in base pairs.
(TIF)

Figure S6 **Co-localization of CysPc domain and H49 degenerate repeats in cytoskeletal fractions.** Cytoskeleton fractions of epimastigotes (CL Brener) were obtained by lysis with Nonidet P40 (0.5%), fixed with 2% paraformaldehyde and incubated with rabbit anti-CysPc, mouse anti-H49 deg antibodies followed by anti-rabbit IgG-FITC (upper left panel, in green), anti-mouse IgG- Alexa 568 (upper right panel, in red) secondary antibodies. The lower panels present the corresponding phase-contrast image and the merged image of the three fluorescence channels (including DAPI, in blue). Scale bar, 5 µm.
(TIF)

Figure S7 **H49 repeats and the FAZ structure (monoclonal antibody 4D9) in whole parasite cells.** Epimastigotes (CL Brener) were permeabilized with Triton X-100 (0.1%), fixed with 2% paraformaldehyde and incubated with rabbit anti-H49 and monoclonal anti-4D9 antibodies. DNA was stained with DAPI (blue). Primary antibodies were revealed using anti-rabbit IgG-Texas Red (upper left, in red) and anti-mouse IgG-FITC (upper right panel, green) secondary antibodies. The lower panels present the corresponding phase-contrast image and the merged images of the fluorescence channels and DAPI (in blue). Scale bar, 5 µm.
(TIF)

Table S1 **H49/calpain genes identified in the genome by tblastn.**
(XLS)

Table S2 **Oligonucleotides used in cloning and RT-PCR experiments.**
(XLS)
