# Supplementary figures and images for: The Repetitive Cytoskeletal Protein H49 of Trypanosoma cruzi Is a Calpain-Like Protein Located at the Flagellum Attachment Zone
Source: PLoS One. 2011 Nov 11;6(11):e27634. doi: 10.1371/journal.pone.0027634 (PMC3214072; doi:10.1371/journal.pone.0027634)

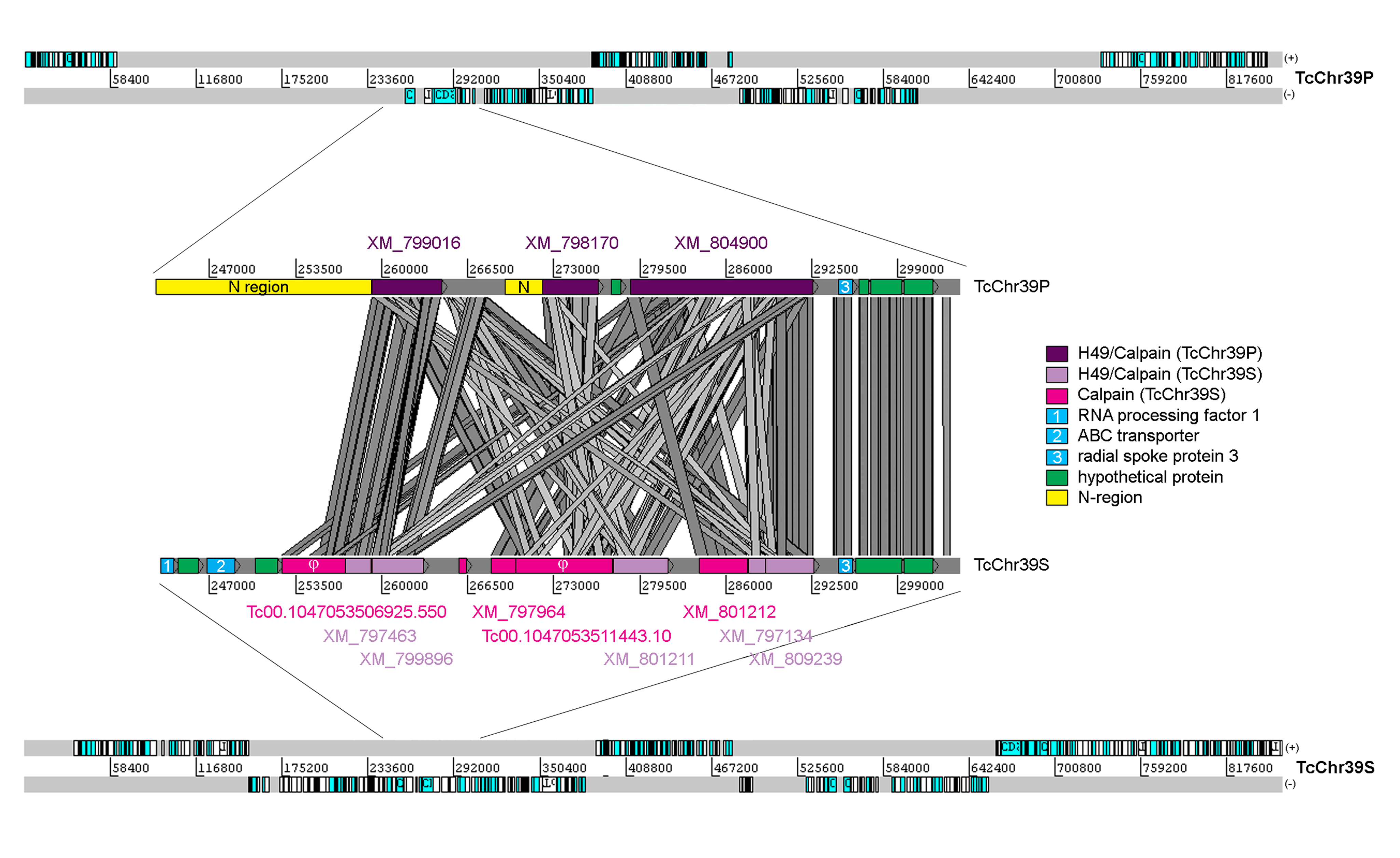

Supplement: Figure S1 — Schematic overview of the genomic regions containing the H49/calpain genes on the T. cruzi chromosome sized scaffolds TcChr39-P and -S. Arrangement of the H49/calpain gene family in chromosomal clusters TcChr39-P and -S (“S” chromosome assigned to the Esmeraldo haplotype and “P” to the non-Esmeraldo haplotype). Comparison between regions from TcChr39-P and -S containing H49/calpains. Homologous genes are connected by gray lines. H49/calpain genes belonging to TcChr39-P and -S are represented by dark and light purple rectangles, respectively. Sequences deposited in the T. cruzi database as calpain-like are indicated by pink rectangles. Locus names are written below each rectangle. The symbol ϕ indicates a pseudogene. Green rectangles represent hypothetical proteins and numbers 1, 2 and 3 inside blue rectangles correspond to RNA processing factor 1, ABC transporter and radial spoke protein 3, respectively. N regions (nucleotide not determined) are indicated by yellow blocks marked by the letter N. Above and below the alignment are regions of TcChr39-P and TcChr39-S, respectively. Genes are drawn in sense strand (signals +) and antisense strand (signal -). (TIF) [file pone.0027634.s001.tif]

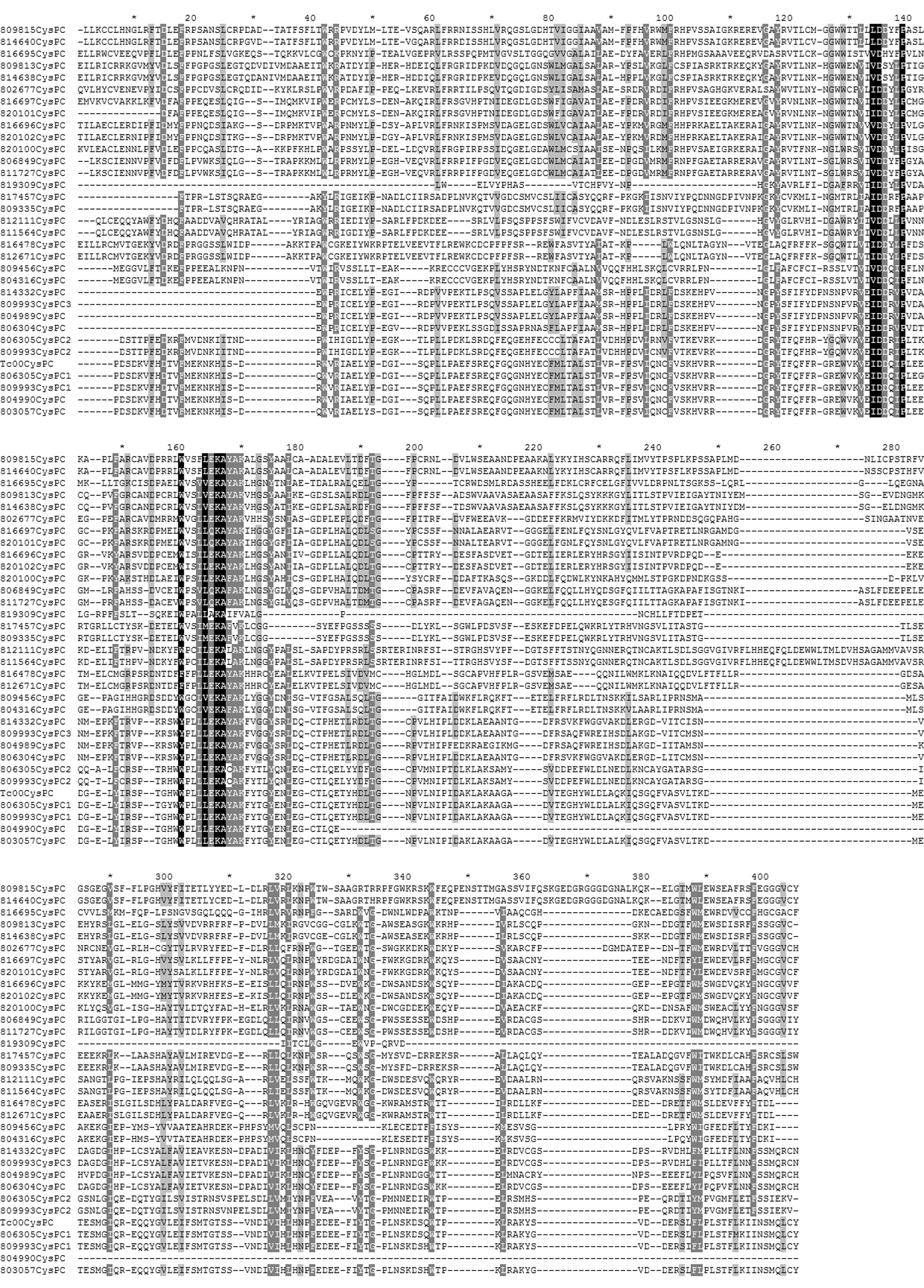

Supplement: Figure S2 — Multiple alignment of the CysPc domain of T. cruzi calpain-like proteins. Identical residues are highlighted in black; dark gray, 80% identity; and light gray, 60% identity. The accession numbers of amino acid sequences are indicated on the right, omitting the initial letters XP. Sequences appear in the same order as they appeared in the phylogenetic tree (Figure 2). In this figure, the CysPc domain of the pseudogene Tc00.1047053506925.550 was included and is indicated by Tc00CysPc. Sequences XP_809993 and XP_806305 have three and two CysPc domains, respectively, which are indicated by numbers 1, 2 and 3. (TIF) [file pone.0027634.s002.tif]

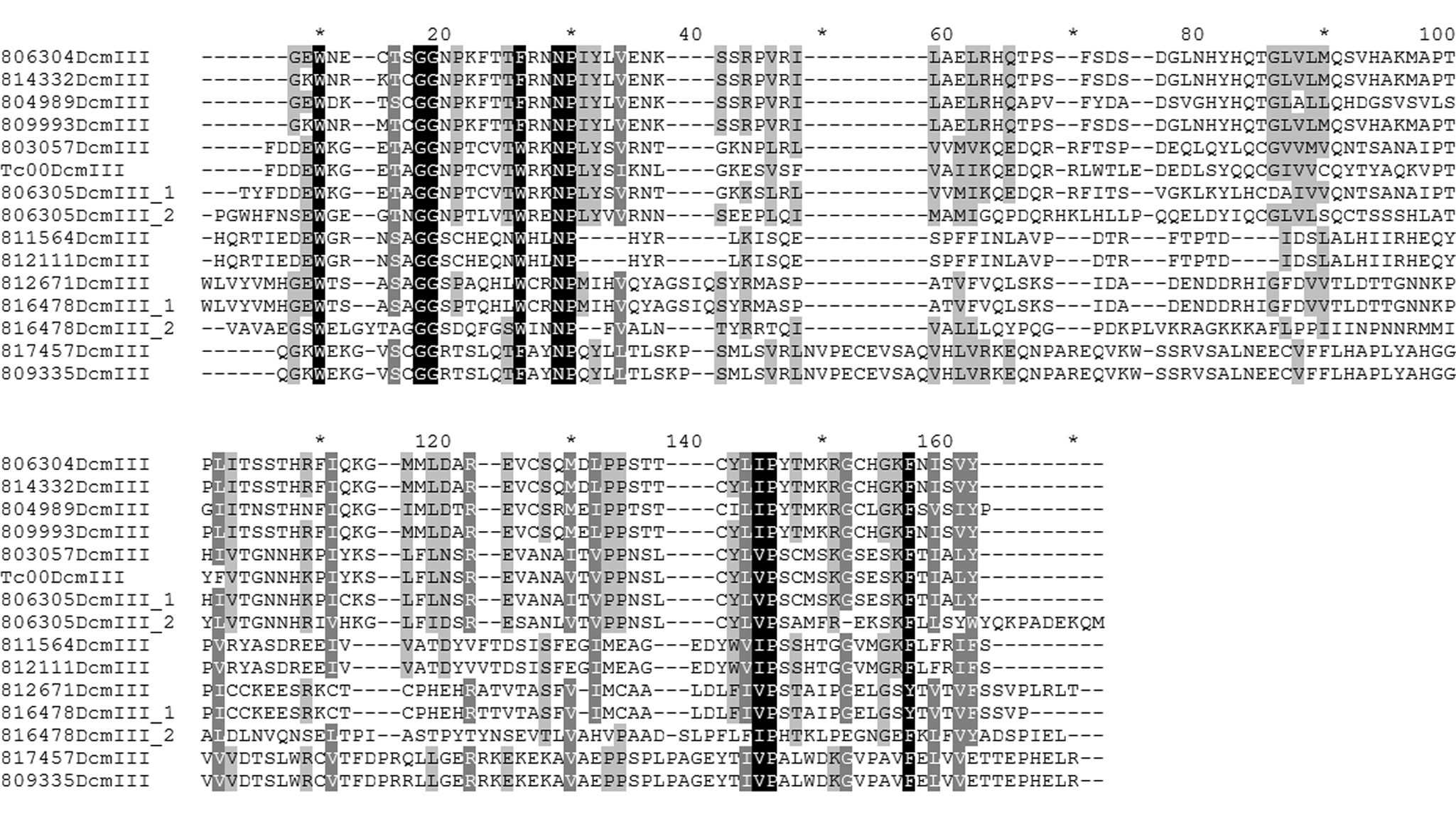

Supplement: Figure S3 — Multiple alignment of the domain III of T. cruzi calpain-like proteins. Identical residues are highlighted in black; dark gray, 80% identity; and light gray, 60% identity. The accession numbers of amino acid sequences are indicated on the right, omitting the initial letters XP. (TIF) [file pone.0027634.s003.tif]

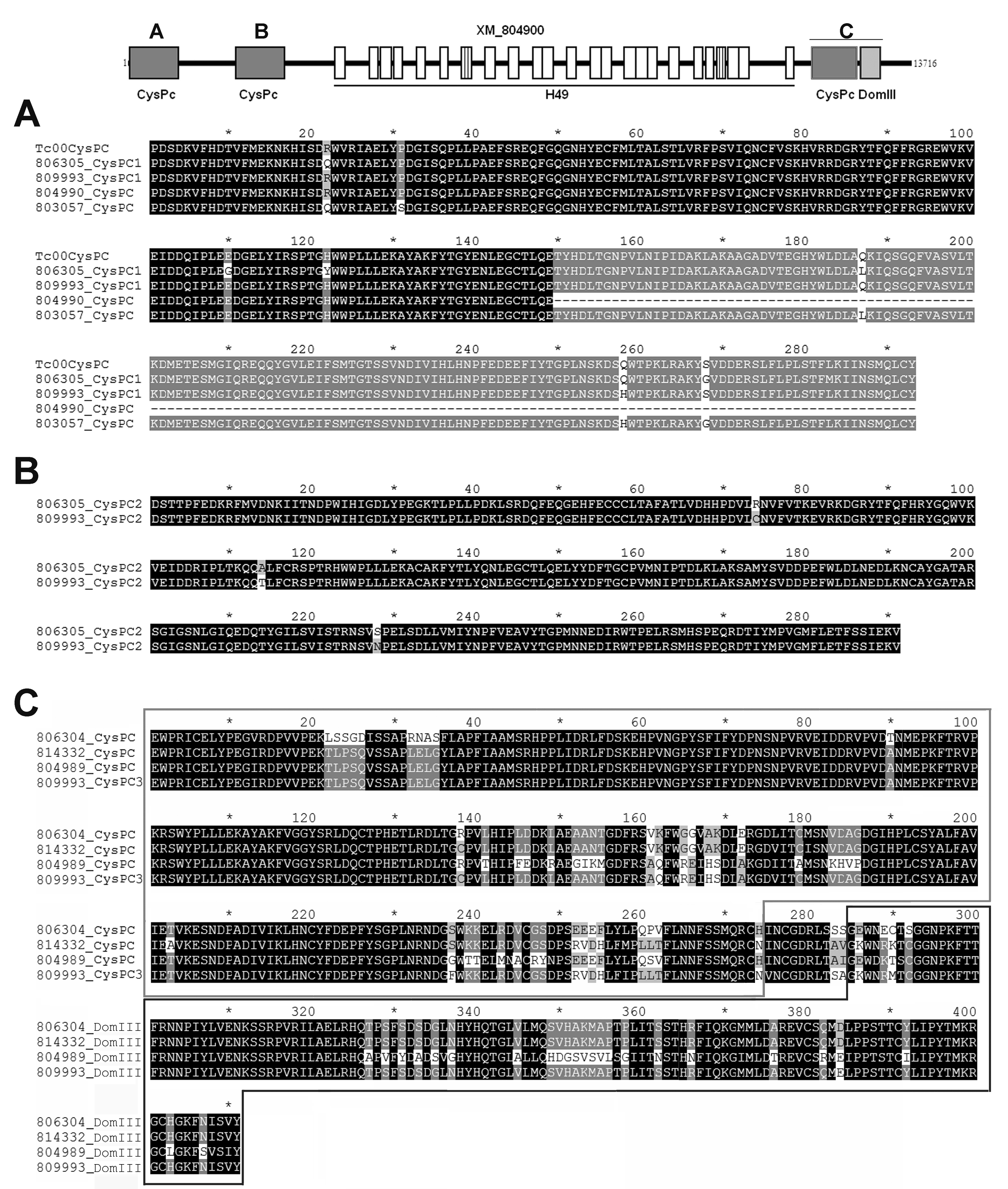

Supplement: Figure S4 — Alignment of the first, second and third domains CysPc of H49/calpains. CysPc domains of H49/calpains were grouped in different arms of the phylogenetic tree (Figure 2). According to the position in chromosomes TcChr39-P and -S, the complete sequences of these calpains were predicted (Figure 4). The first (A), second (B) and third (C) CysPc domains were aligned using the ClustalW method. At letter C, the third CysPc domains are boxed in red and domain III are boxed in blue. Identical residues are highlighted in black; dark gray, 80% identity; and light gray, 60% identity. The accession numbers of amino acid sequences are indicated on the right, omitting the initial letters XP. Sequences XP_809993 and XP_806305 have three and two CysPc domains, respectively, which are indicated by numbers 1, 2 and 3. (TIF) [file pone.0027634.s004.tif]

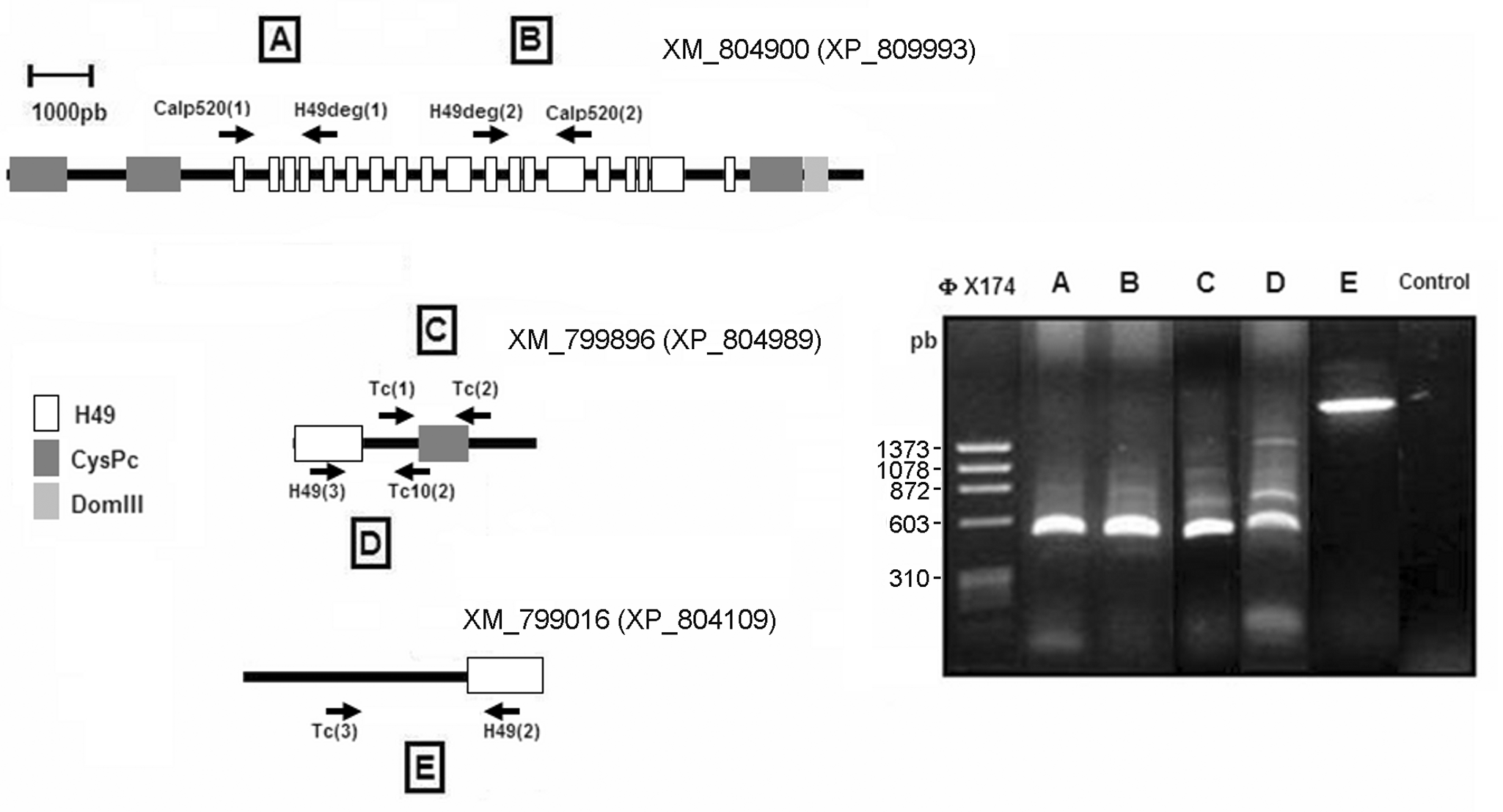

Supplement: Figure S5 — RT-PCR amplification of H49/calpain sequences from epimastigotes (CL Brener). Left) The primers were based on the sequence of H49 repeats and catalytic domain (CysPc) from H49/calpains indicated in the figure. The regions A and B containing the degenerate H49 repeat of gene XM_804900 (XP_809993) were amplified using the primers Calp520(1) and H49deg (1) and H49deg(2) and Calp520(2), respectively. The regions C and D from gene XM_799896 (XP_804989), containing the catalytic domain of calpain (CysPc) and the CysPc and H49, were amplified using the primers Tc(1) and Tc(2), and H49(3) and Tc10(2). The region D from gene XM_799016 (XP_804109) was amplified using the primers Tc(3) and H49(2). Right) Electrophoresis on agarose gels demonstrating the amplicons carrying the various regions (A–E) of H49/calpain genes. The control was carried out with total epimastigote RNA treated with DNase. The molecular size markers were indicated in base pairs. (TIF) [file pone.0027634.s005.tif]

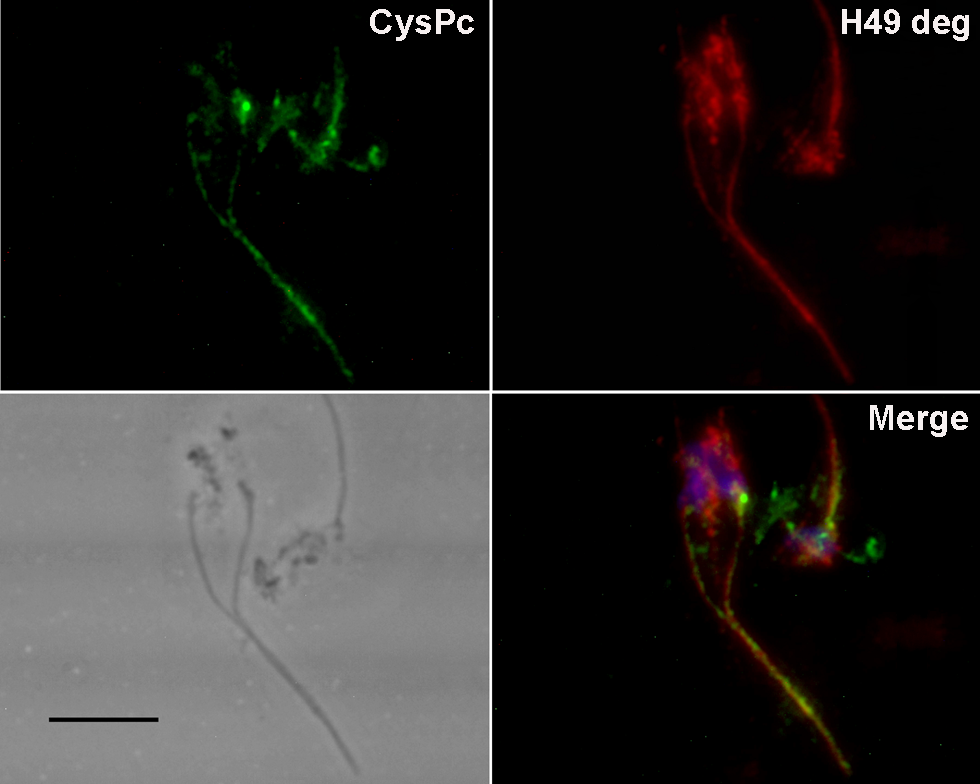

Supplement: Figure S6 — Co-localization of CysPc domain and H49 degenerate repeats in cytoskeletal fractions. Cytoskeleton fractions of epimastigotes (CL Brener) were obtained by lysis with Nonidet P40 (0.5%), fixed with 2% paraformaldehyde and incubated with rabbit anti-CysPc, mouse anti-H49 deg antibodies followed by anti-rabbit IgG-FITC (upper left panel, in green), anti-mouse IgG- Alexa 568 (upper right panel, in red) secondary antibodies. The lower panels present the corresponding phase-contrast image and the merged image of the three fluorescence channels (including DAPI, in blue). Scale bar, 5 µm. (TIF) [file pone.0027634.s006.tif]

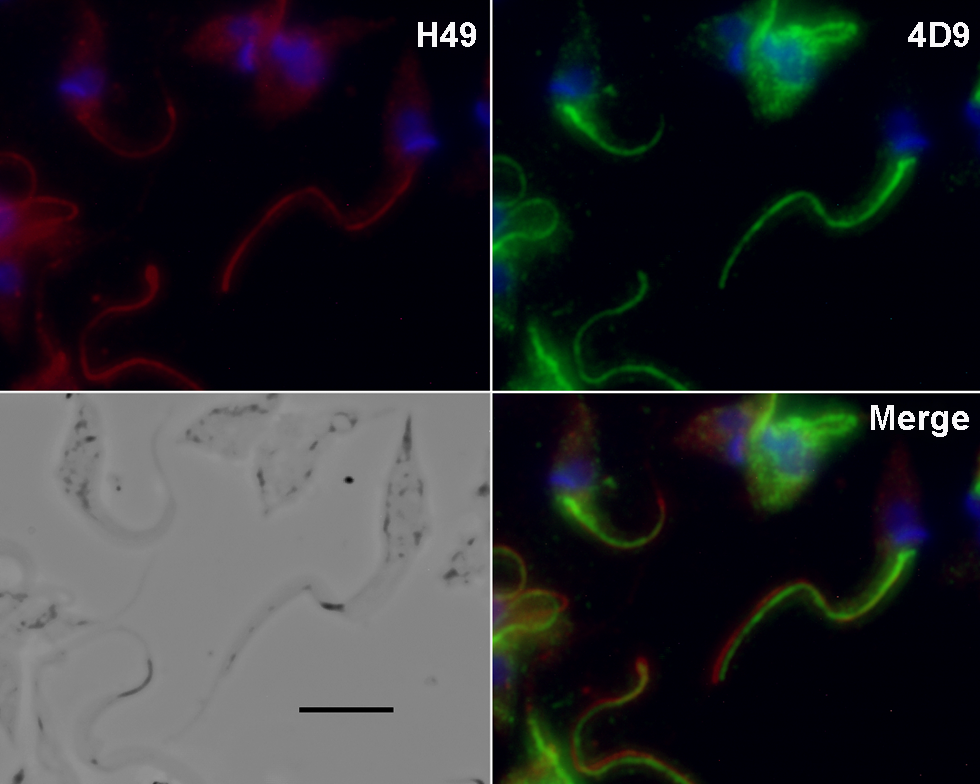

Supplement: Figure S7 — H49 repeats and the FAZ structure (monoclonal antibody 4D9) in whole parasite cells. Epimastigotes (CL Brener) were permeabilized with Triton X-100 (0.1%), fixed with 2% paraformaldehyde and incubated with rabbit anti-H49 and monoclonal anti-4D9 antibodies. DNA was stained with DAPI (blue). Primary antibodies were revealed using anti-rabbit IgG-Texas Red (upper left, in red) and anti-mouse IgG-FITC (upper right panel, green) secondary antibodies. The lower panels present the corresponding phase-contrast image and the merged images of the fluorescence channels and DAPI (in blue). Scale bar, 5 µm. (TIF) [file pone.0027634.s007.tif]
